# Emergency department visits and trends related to cocaine, psychostimulants, and opioids in the United States, 2008–2018

**DOI:** 10.1186/s12873-022-00573-0

**Published:** 2022-02-04

**Authors:** Leslie W. Suen, Thibaut Davy-Mendez, Kathy T. LeSaint, Elise D. Riley, Phillip O. Coffin

**Affiliations:** 1 National Clinician Scholars Program, Philip R. Lee Institute for Health Policy Studies, University of California San Francisco, 290 Illinois Street, Suite 7227, Box 0936, San Francisco, CA 94158 USA; 2 San Francisco Veterans Affairs Medical Center, San Francisco, CA USA; 3 Division of Infectious Diseases, Department of Medicine, University of North Carolina, Chapel Hill, NC USA; 4 Department of Emergency Medicine, University of California, San Francisco, San Francisco, CA USA; 5 Department of Medicine, Division of HIV, Infectious Diseases and Global Medicine, School of Medicine, University of California, San Francisco, San Francisco, CA USA; 6 Department of Public Health, San Francisco, CA USA

**Keywords:** Amphetamines, Cocaine, Stimulants, Emergency department, Opioids, Overdose

## Abstract

**Background:**

Drug-related emergency department (ED) visits are escalating, especially for stimulant use (*i.e.,* cocaine and psychostimulants such as methamphetamine). We sought to characterize rates, presentation, and management of ED visits related to cocaine and psychostimulant use, compared to opioid use, in the United States (US).

**Methods:**

We used 2008–2018 National Hospital Ambulatory Medical Care Survey data to identify a nationally representative sample of ED visits related to cocaine and psychostimulant use, with opioids as the comparator. To make visits mutually exclusive for analysis, we excluded visits related to 2 or more of the three possible drug categories. We estimated annual rate trends using unadjusted Poisson regression; described demographics, presenting concerns, and management; and determined associations between drug-type and presenting concerns (categorized as psychiatric, neurologic, cardiopulmonary, and drug toxicity/withdrawal) using logistic regression, adjusting for age, sex, race/ethnicity, and homelessness.

**Results:**

Cocaine-related ED visits did not significantly increase, while psychostimulant-related ED visits increased from 2008 to 2018 (2.2 visits per 10,000 population to 12.9 visits per 10,000 population; *p* < 0.001). Cocaine-related ED visits had higher usage of cardiac testing, while psychostimulant-related ED visits had higher usage of chemical restraints than opioid-related ED visits. Cocaine- and psychostimulant-related ED visits had greater odds of presenting with cardiopulmonary concerns (cocaine adjusted odds ratio [aOR] 2.95, 95% CI 1.70–5.13; psychostimulant aOR 2.46, 95% CI 1.42–4.26), while psychostimulant-related visits had greater odds of presenting with psychiatric concerns (aOR 2.69, 95% CI 1.83–3.95) and lower odds of presenting with drug toxicity/withdrawal concerns (aOR 0.47, 95%CI 0.30–0.73) compared to opioid-related ED visits.

**Conclusion:**

Presentations for stimulant-related ED visits differ from opioid-related ED visits: compared to opioids, ED presentations related to cocaine and psychostimulants are less often identified as related to drug toxicity/withdrawal and more often require interventions to address acute cardiopulmonary and psychiatric complications.

**Supplementary Information:**

The online version contains supplementary material available at 10.1186/s12873-022-00573-0.

## Introduction

Emergency department (ED) visits related to drug overdose are escalating, especially during the COVID-19 pandemic [1]. While national attention has focused on opioids in the escalating overdose crisis, EDs across the United States (US) are also facing an expanding burden of visits related to stimulant use [2–5]. Stimulant-related ED visits are largely attributed to the use of substances such as cocaine and psychostimulants (e.g., methamphetamine) [6–9]. Cocaine has contributed to stimulant-related harms since at least 2011 [9], though in recent years, the US has seen a surge in methamphetamine use and related complications [10]. In addition to stimulant-related ED visits increasing nationwide [5], stimulant-related overdose deaths have also rapidly increased since 2010 [11] and now outnumber deaths attributed to prescription opioids and heroin [8].

Prior studies have mostly focused on ED visits related to stimulant overdose [1, 4, 5]. However, acute stimulant toxicity is not always recognized as an overdose [12], and these studies do not address visits related to chronic complications of drug use. There is a need to broadly examine the characteristics, presentations, and clinical management of all stimulant-related ED visits, not only visits that are identified as related to overdose. Presentations from stimulant toxicity also show wide variability, affect multiple organ systems, and are not as readily attributed to substance use [4, 13–16]. Cocaine toxicity is strongly associated with cardiovascular complications [13, 17], while psychostimulant toxicity is also associated with cardiac complications, as well as acute psychosis and agitation [3, 4, 14]. Few studies elucidate the prevalence of presenting concerns for psychostimulant-related ED visits or how they may vary compared to opioid-related ED visits. A better understanding of how patients with stimulant-related concerns present to the ED is needed, as this information has significant implications on clinical management and ED resource utilization.

We sought to describe the annual trends and characteristics of cocaine- and psychostimulant-related ED visits, with opioids-related ED visits as the reference group, given epidemiology and presentations of opioid-related visits are well understood and elucidated. We also evaluated whether chief presenting concerns during ED visits differ between these groups of drug-related ED visits.

## Methods

### Study design and setting

We conducted a secondary analysis using 2008–2018 data from the National Hospital Ambulatory Medical Care Survey (NHAMCS), a nationally representative dataset of ED visits collected annually by the Centers for Disease Control and Prevention National Center for Health Statistics (NCHS) [18]. Each year, trained staff abstract visit data from medical records using standardized recording forms, including demographics, reasons for visit (RFV), diagnostic testing, administered medications, diagnoses rendered, etc. NHAMCS uses a multi-stage probability design and applies survey weights to obtain a nationally representative sample of ED visits to non-federal, general, short-stay acute care US hospitals [18, 19].

For each ED visit in NHAMCS in 2008–2013, ≤ 3 associated diagnosis codes, ≤ 3 RFV codes, and ≤ 8 administered medications were available [20, 21]. For 2014–2018, ≤ 5 associated diagnosis codes, ≤ 5 RFV codes, and ≤ 30 administered medications were available [22]. For consistency, we used only the top three listed diagnosis codes, top three listed RFV codes as their chief presenting concerns, and top eight listed administered medications.

### Selection of emergency department visits

The sample included ED visits made by adults (≥ 18 years old) with the visit related to cocaine use, psychostimulant use, or opioid use, based on listed *International Classification of Diseases Clinical Modification (ICD-CM)* visit diagnosis codes. We included any visit that had any *ICD-9-CM/ICD-10-CM* code related to drug dependence, abuse, or poisoning for either cocaine, psychostimulant, or opioid use in any of the top three listed diagnoses codes (see Additional file 1 for complete list of *ICD*codes used) [23, 24]. We excluded “in remission” codes to capture visits related to active drug use. We adapted this approach from the Centers for Medicare and Medicaid Services and other studies [2, 4, 25]. To make groups mutually exclusive and allow for comparisons, we excluded visits involving two or more of the three drug-related diagnosis groups (cocaine, psychostimulant, or opioid), approximately 5% of unweighted eligible visits. We excluded visits identified by NHAMCS as follow up visits and visits where the patient was seen at the same hospital within the preceding 72 h to limit repeat visits for the same illness episode.

### Measurements and outcomes

The primary exposure of interest was the type of drug associated with the ED visit, as defined above (cocaine, psychostimulant, or opioid). The primary outcomes of interest were the chief presenting concerns. We sought to determine if presentations of drug-related visits were more commonly identified as belonging to a specific organ system (e.g., psychiatric, neurologic, or cardiopulmonary), or related to the broader constructs of drug toxicity and withdrawal. We measured four separate outcomes of dichotomous variables if visit concerns were related to: (1) psychiatric; (2) neurologic; (3) cardiopulmonary; or (4) drug toxicity/withdrawal concerns. Visits could contribute to more than one category of presenting concerns. For chief presenting concerns, NHAMCS uses an RFV coding scheme, where RFV is defined as “the patient’s complaint(s), symptom(s), or other reasons for this visit.” [20] We adapted the RFV coding scheme to measure if any of the top three RFV codes were related to psychiatric, neurologic, cardiopulmonary, and drug toxicity/withdrawal categories [26]. For example, psychiatric presenting concerns included RFV codes such as *“depression”* and “*suicide attempt*.” Cardiopulmonary concerns included codes such as *“chest pain,” “shortness of breath,”* and *“respiratory arrest.”* Drug toxicity/withdrawal concerns included *“drug detoxification,” “accidental poisoning,”* and *“adverse effects of drug use”* such as unintentional overdose (see Additional file 2 for RFV code categorization).

For each category of drug-related visits (cocaine, psychostimulant, or opioid), we described patient characteristics including age, sex, race/ethnicity, primary payer, homelessness, and multimorbidity (defined as the presence of two or more comorbidities assessed by NHAMCS) [27]. We used the imputed measure of race/ethnicity provided by NHAMCS, which adjusts for 16–18% missingness of race/ethnicity data extracted from the medical chart [21, 22]. We assessed hospital-level factors including urban location, US census region, and safety-net status, defined per NCHS criteria as having either > 30% of visits with the primary payer being Medicaid or uninsured, or having > 40% of visits from combined Medicaid and uninsured [28].

To evaluate characteristics of clinical management, we assessed diagnostic testing, administered medications, and disposition. As above, we analyzed the top eight administered medications, using the drug classification scheme developed by NCHS [21, 29]. We reviewed drug codes available and included any drug codes that could be classified as *“atypical antipsychotics,” “benzodiazepines,” “naloxone,”* or “*opioids*.” As certain combinations of polysubstance use are common (e.g., cocaine and alcohol [30]), we additionally examined whether visits were concurrently associated with alcohol-related and other drug-related diagnoses, including cannabis, sedatives/hypnotics, etc. (Additional file 1).

### Statistical analysis

We applied survey sample weights to yield an unbiased national estimate of ED visit percentages and characteristics [19]. We calculated annual ED visit rates by dividing the weighted number of visits each year by the US Census Bureau estimates of civilian, noninstitutionalized adults aged 18 and older [19]. We conducted a Poisson regression analysis using visit year as the ordinal predictor to test for significant trends in visits over time. We described characteristics of cocaine-, psychostimulant-, and opioid-related visits using bivariate Pearson chi-squared tests. We used multivariable logistic regression to evaluate whether type of drug-related visit was associated with specific categories of chief presenting concerns, using separate models for each category. We adjusted for age, sex, race/ethnicity, and homelessness, as these factors could be associated with increased morbidity. All reported estimates were robust, defined by NCHS as an unweighted count in each group of ≥ 30 visits and/or a relative standard error of ≤ 30% [18, 31]. We completed all analyses using *svy*routine commands within Stata/MP, version 16.0 (StataCorp LLC) [19, 32]. All *p*-values were two-sided and a *p*-value < 0.05 was used to determine statistical significance. The University of California, San Francisco Institutional Review Board exempted this study from review.

## Results

### Annual rate trends of drug-related emergency department visits

**Fig. 1:**
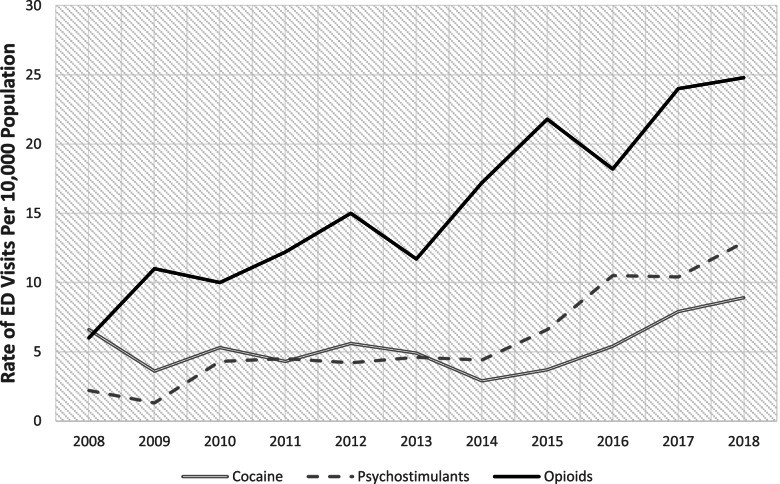
Annual trends in rates of national emergency department visits related to cocaine, psychostimulant, or opioid use, 2008–2018. Emergency department visits categorized by drug-type if any of the top three *ICD9-CM/ICD10-CM* diagnoses codes were related to opioid, cocaine, or psychostimulant use. Visits were mutually exclusive for drug type, as visits associated with two or more drug-categories were excluded. Rates were calculated by dividing weighted number of visits in each year by US Census Bureau estimates of civilian, noninstitutionalized adults aged 18 and older for that year. All rates per 10,000 population. Source: National Hospital Ambulatory Medical Care Survey

The study sample included 1,576,000 unweighted ED visits between 2008 to 2018, which was representative of 7,121,000 weighted ED visits. The rate of cocaine-related ED visits increased from 6.6 visits per 10,000 population (95%CI 3.9–9.3) in 2008 to 8.9 visits per 10,000 population (95%CI 4.7–13.1) in 2018, though this was not statistically significant (*p* = 0.23). Rates of psychostimulant-related ED visits increased from 2.2 visits per 10,000 population (95%CI 0.8–3.7) to 12.9 visits per 10,000 population (95%CI 7.3–18.4) (*p* < 0.001). The increase in ED visits was greatest for opioids, where rates of opioid-related ED visits increased from 6.0 visits per 10,000 population (95%CI 3.7–8.2) in 2014 to 24.8 visits per 10,000 population (95%CI 18.0–31.5) in2018 (*p* < 0.001) (Fig. **1**).

### Characteristics of drug-related emergency department visits

In unadjusted analysis, psychostimulant- and opioid-related visits had comparable age and race/ethnicity distributions, whereas cocaine-related visits were more likely to be among those ≥ 40 years of age and identifying as Black (*p* < 0.001 for both characteristics) (Table **1**). Patients with cocaine- and psychostimulant-related visits were more likely to be experiencing homelessness compared to patients with opioid-related visits (cocaine 10%, psychostimulant 12%, opioid 4%; *p* < 0.001). Cocaine-related visits were predominantly in the South (49%), while psychostimulant-related visits were more concentrated in the West (60%), and opioid-related visits were spread out throughout all regions. Cocaine and psychostimulant-related visits were slightly more concentrated at hospitals in urban areas than opioid-related visits (cocaine 96%, psychostimulant 91%, opioid 88%; *p* = 0.03).

**Table 1:** Demographic characteristics of national emergency department visits related to cocaine, psychostimulant, opioid use, 2008–2018

	**Weighted % (95% CI)**		
	**Cocaine-related visits** **(*n* = 1,406,000)**	**Psychostimulant-related visits** **(*n* = 1,590,000)**	**Opioid-related visits** **(*n* = 4,125,000)**
**Age, years**			
18–29	12 (9, 17)	38 (31, 46)	38 (33, 43)
30–39	26 (19, 35)	31 (24, 40)	26 (22, 30)
40–49	29 (23, 37)	14 (10, 21)	13 (10, 16)
≥ 50	32 (25, 40)	16 (10, 24)	24 (20, 29)
**Female**	39 (33, 46)	39 (32, 47)	47 (42, 51)
**Race/ethnicity**			
Black	54 (45, 62)	10 (7, 15)	11 (8, 15)
White	28 (22, 36)	64 (55, 72)	78 (73, 82)
Other race/ethnicity	18 (13, 25)	26 (19, 35)	11 (8, 15)
**Primary Payer**			
Medicaid	28 (22, 34)	36 (27, 45)	34 (29, 40)
Medicare	13 (7, 23)	5 (3, 8)	13 (10, 17)
Private	16 (11, 23)	19 (13, 26)	19 (15, 23)
Uninsured	24 (19, 30)	24 (17, 34)	17 (14, 20)
Other	19 (13, 27)	16 (10, 26)	17 (12, 23)
**Homelessness**	10 (6, 17)	12 (8, 17)	4 (2, 6)
**Urban region**	96 (92, 98)	91 (83, 96)	88 (82, 93)
**US Region**			
Northeast	16 (11, 23)	–	22 (18, 26)
Midwest	20 (14, 26)	–	24 (20, 29)
South	49 (40, 59)	28 (22, 36)	27 (23, 33)
West	15 (10, 21)	60 (51, 68)	27 (21, 32)
**Safety Net Hospital**	28 (22, 36)	26 (19, 35)	24 (20, 29)
**Multimorbidity**	19 (13, 28)	–	13 (10, 17)

Source: National Hospital Ambulatory Medical Care Survey. Cell sizes with < 30 unweighted visits or > 30% relative standard error not included. Visits were mutually exclusive for drug type, as visits associated with two or more drug-categories were excluded. Multimorbidity as the presence of two or more comorbidities assessed by NHAMCS (including Alzheimer’s dementia, alcohol use disorder, asthma, cancer, cerebrovascular disease, chronic obstructive pulmonary disease, coronary artery disease, depression, diabetes, chronic kidney disease, end stage renal disease, venous thromboembolism, HIV/AIDS, hypertension, obesity, obstructive sleep apnea, osteoporosis, and substance use disorders)

Psychiatric concerns were more common in cocaine- and psychostimulant-related visits (cocaine 31%, psychostimulant 50%, opioid 25%; *p* < 0.001). Cardiopulmonary concerns were also more common in cocaine- and psychostimulant-related visits (cocaine 33%, psychostimulant 23%, opioid 12%; *p* < 0.001) concerns than opioid-related visits (Table **2**). Drug-toxicity/withdrawal concerns were more common in opioid-related visits (cocaine 36%, psychostimulant 32%, opioid 49%; *p* < 0.001). Cocaine-related visits had a higher proportion of co-occurring alcohol-related diagnoses in the same ED visit (cocaine 19%, psychostimulant 6%, opioid 7%; *p* < 0.001).

**Table 2:** Presenting concerns, clinical management, and disposition of national emergency department visits related to cocaine, psychostimulant, or opioid use, 2008–2018

	**Weighted % (95% CI)**		
	**Cocaine-related visits** **(*n* = 1,406,000)**	**Psychostimulant-related visits** **(*n* = 1,590,000)**	**Opioid-related visits** **(*n* = 4,125,000)**
**Chief presenting concern(s)**			
Psychiatric	31 (24, 38)	50 (42, 58)	25 (21, 30)
Neurologic	7 (4, 11)	7 (4, 12)	7 (4, 10)
Cardiopulmonary	33 (26, 41)	23 (17, 31)	12 (10, 16)
Drug toxicity/withdrawal	36 (30, 43)	32 (25, 40)	49 (43, 54)
**Co-occurring Drug Related Diagnoses**			
Alcohol-related diagnosis	19 (15, 25)	6 (4, 10)	7 (5, 9)
Other drug-related diagnosis	9 (6, 13)	9 (6, 14)	9 (7, 12)
**Diagnostic testing**			
Blood alcohol concentration	20 (15, 27)	20 (14, 29)	16 (12, 19)
Cardiac monitoring	24 (18, 32)	13 (8, 20)	12 (9, 16)
Cardiac biomarkers	23 (16, 32)	11 (7, 16)	7 (5, 9)
Electrocardiogram	51 (43, 59)	34 (27, 41)	29 (24, 33)
Urine toxicology	56 (47, 64)	42 (34, 51)	35 (30, 41)
Any imaging	54 (46, 61)	35 (27, 44)	35 (30, 41)
Any X-ray	44 (37, 52)	28 (21, 37)	26 (20, 31)
Any CT Scan	17 (12, 23)	14 (8, 21)	13 (10, 17)
**Administered medications**			
Atypical antipsychotics	6 (4, 11)	13 (8, 20)	2 (1, 3)
Benzodiazepines	19 (14, 25)	33 (26, 42)	15 (12, 19)
Naloxone	–	–	13 (10, 17)
Opioids	17 (11, 24)	9 (4, 17)	14 (11, 18)
**Disposition**			
Treat and release	58 (51, 65)	63 (55, 70)	68 (63, 72)
Left before treatment complete	3 (1, 5)	2 (1, 6)	3 (2, 6)
Transferred to psychiatric facility	6 (3, 13)	10 (6, 17)	5 (3, 7)
Admitted	16 (12, 22)	9 (6, 13)	16 (13, 20)

Source: National Hospital Ambulatory Medical Care Survey. Cell sizes with < 30 unweighted visits or > 30% relative standard error not included. Visits were mutually exclusive for drug type, as visits associated with two or more drug-categories were excluded. Chief presenting concerns defined using top three “reason for visit” codes. Visits could contribute to more than one category of chief presenting concerns

The most common chief presenting concerns varied across groups. The most common chief presenting concern for opioid-related visits were *“adverse effect of drug abuse”* (27.9% of opioid-related visits), *“drug detoxification”* (6.0%), and *“abnormal drug usage”* (5.7%) (Additional file 3). The most common chief presenting concerns for cocaine-related visits were *“chest pain”* (27.2% of cocaine-related visits), *“other problems relating to psychosis”* (7.9%), and “*abdominal pain*” (5.7%). For psychostimulant-related visits, most common chief presenting concerns were *“chest pain”* (10.4% of psychostimulant-related visits), “*abnormal drug usage*” (9.3%), and “*other symptoms related to psychosis*” (8.7%).

Cocaine-related visits had the highest utilization of diagnostic testing, especially cardiovascular testing (e.g., cardiac biomarkers and monitoring). Psychostimulant-related visits had more administration of chemical restraint medications. Cocaine- and opioid-related visits more often resulted in admission (cocaine: 16%, psychostimulant 9%, opioid 16%; *p* < 0.001), while psychostimulant-related visits had more transfers to psychiatric facilities (cocaine 6%, psychostimulant 10%, opioid 5%; *p* < 0.001).

### Multivariable analyses

Unadjusted odd ratios (OR) showing associations between drug type and presenting concerns are shown in Table 3. Adjusting for age, sex, race/ethnicity, and homelessness, psychostimulant-related visits had greater odds of presenting with a psychiatric chief concern compared to opioid-related visits (adjusted odds ratio [aOR] 2.69; 95% CI 1.83–3.95; Table 3). No differences were seen with neurologic chief concerns. Both cocaine- and psychostimulant-related visits had greater odds of presenting with cardiopulmonary chief concerns compared to opioid-related visits (cocaine aOR 2.95, 95% CI 1.70–5.13: psychostimulant aOR 2.46, 95% CI 1.42–4.27). In contrast, psychostimulant-related visits had lower odds of presenting with drug toxicity/withdrawal concerns (aOR 0.47, 95%CI 0.30–0.73).

**Table 3:** Associations between drug type and chief presenting concerns among national emergency department visits related to cocaine, psychostimulant, or opioid use, 2008–2018

	**Psychiatric chief concerns**		**Neurologic chief concerns**		**Cardiopulmonary chief concerns**		**Drug toxicity/ withdrawal chief concerns**	
	**OR**	**95%CI**	**OR**	**95%CI**	**OR**	**95%CI**	**OR**	**95%CI**
***Unadjusted analyses***								
** Drug**								
Cocaine	1.32	0.85–2.21	0.99	0.51–1.95	3.52	2.34–5.31	0.60	0.42–0.87
Psychostimulants	2.99	2.04–4.39	1.03	0.49–2.17	2.12	1.26–3.58	0.49	0.32–0.76
Opioid	Ref		Ref		Ref		Ref	
***Adjusted analyses***								
** Drug**								
Cocaine	1.37	0.85–2.21	1.05	0.87–2.28	2.95	1.70–5.13	0.83	0.52–1.35
Psychostimulants	2.69	1.83–3.95	0.92	0.36–2.37	2.46	1.42–4.26	0.47	0.30–0.73
Opioid	Ref		Ref		Ref		Ref	

Source: National Hospital Ambulatory Medical Care Survey. Visits were mutually exclusive for drug type, as visits associated with two or more drug-categories were excluded. Chief presenting concerns defined using top three “reason for visit” codes. Visits could contribute to more than one category of chief presenting concerns. Adjusted analyses were adjusted for age, sex, race/ethnicity, and homelessness. *OR* Odds Ratio, *CI* confidence interval

## Discussion

In this study using nationally representative ED visits, there were significant increases in psychostimulant-related ED visits, and presentations for both cocaine- and psychostimulant-related ED visits differed compared to opioid-related ED visits.

Psychostimulant-related ED visits increased from 2.2 to 12.9 visits per 10,000 population from 2008 to 2018. This is consistent with studies showing increasing national rates of ED visits, hospitalizations, and deaths from psychostimulant overdose [2, 4, 5, 33]. The increasing use of the ED and other acute care settings is likely linked to rising methamphetamine availability and use [34]. National Forensic Laboratory Information System data found methamphetamine case submissions increased from 2011 to 2019, with methamphetamine as the most frequently reported drug [35]. While psychostimulant-related ED visits were predominantly among Western regions in our study, recent data highlights the emergence of psychostimulant-related overdose deaths in the Midwest and Northeast, suggesting methamphetamine is already a nationwide concern [8, 36]. Increases in cocaine-related ED visits were not significant, potentially due to the exclusion of visits related to opioid and cocaine co-use. Polysubstance use is common in among individuals using cocaine [30], and other studies found rates of fatal overdoses and ED visits for overdose involving cocaine and opioid use are rising [5, 33].

We found stimulant-related ED visits were less likely to be identified as drug toxicity/withdrawal concerns, underscoring the differences in presentations between stimulant- and opioid-related visits. While the national surge in ED visits and deaths related to opioid overdose is linked to the rise in fentanyl in the drug supply [1, 33, 37], the main drivers of stimulant-related ED visits and overdoses are unclear. Possibilities include increased potency of fluctuating drug supplies [35], contamination or co-use with synthetic opioids like fentanyl [38], or the cumulative effects of chronic stimulant use over time [39]. Further, the term “overdose”, when applied to opioids commonly refers to an acute respiratory event from an episode of use, and this term is problematic when applied to stimulants, as it lacks specificity in capturing the diverse ways in which stimulant toxicity can present [16, 40]. Our data suggest that acute emergency presentations related to stimulant use are more likely due to the cumulative effect of stimulant use over time rather than from a single episode of use. Addressing acute stimulant toxicity may rely more on clinical management of various symptoms, rather than the development of a single reversal agent like naloxone for opioid overdose.

Cocaine-related ED visits were predominately made by individuals who were older, male, and Black. Potential reasons include differences in drug supply, disparities in comorbidities, socioeconomic disadvantage, and other factors related to structural racism that can affect health and healthcare access [41, 42]. Complications from cocaine use are disproportionately higher in Black communities, where rates of cocaine-related deaths are comparable to the rates of opioid-related deaths in white individuals [41]. Yet cocaine-related harms have been understudied in recent years. This is alarming given overdose deaths in Black individuals are rising faster compared to whites [43, 44], and in our study, cocaine-related visits were as likely to result in admission as opioid-related visits. As attention toward the rising epidemic of stimulant-related deaths increases, interventions addressing stimulant use must address racial equity and pay attention to both cocaine and psychostimulant use to avoid further exacerbating racial and economic disparities [45].

Both cocaine- and psychostimulant-related ED visits were associated with cardiopulmonary concerns, even after adjusting for cardiovascular risk factors. Both cocaine and psychostimulants are known cardiotoxins, and both acute and chronic use can lead to adverse events such as myocardial ischemia, stroke, and heart failure [17, 46, 47]. Simultaneous cocaine and alcohol use is also common and contributes to worse cardiovascular outcomes [48, 49]. Patients using stimulants who develop chronic cardiovascular conditions such as heart failure, despite being younger, face more severe in-hospital complications with higher rates of readmission compared to patients with non-stimulant-related heart failure [50, 51]. These findings suggest a need for targeted cardiopulmonary interventions for people who present to the ED with stimulant-related diagnoses.

Psychostimulant-related ED visits were strongly associated with psychiatric concerns, greater administration of chemical restraint medications, and more transfers to psychiatric facilities. Single center ED studies have drawn similar conclusions [14, 15], and our national study adds generalizability. Methamphetamine use not only precipitates psychotic symptoms but also exacerbates underlying psychiatric illness [14, 52]. Paired with high rates of homelessness seen in patients with stimulant-related ED visits, these findings are especially relevant for urban areas where addressing acute psychiatric presentations and homelessness are pressing concerns.

The ED has become a critical setting in responding to the overdose crisis and addressing the health needs of people who use drugs, particularly among low-income populations [53, 54]. EDs have taken up campaigns to distribute naloxone[55–57], initiate buprenorphine prescriptions for opioid use disorder treatment [54], and develop referral programs to increase linkages to treatment [58, 59]. Similarly, the ED can become a point of intervention for addressing acute and chronic complications of stimulant use disorders [60]. While no current Food and Drug Administration approved medications for stimulant use disorder exist, several medications show early promise [61–63]. Behavioral treatments such as contingency management and cognitive behavioral therapy are effective, though few can access these treatment modalities, especially for publicly-insured populations where reimbursement is limited [64–66]. EDs facing a high burden of stimulant-related visits could implement a navigator to facilitate linkages to existing treatment programs [58, 67], or offer harm reduction kits to reduce complications of drug use. As methamphetamine-related ED visits are associated with longer length of stays and higher costs [14], facilitating linkages to stimulant use treatment from the ED could help reduce costs and align with national policies focused on expanding access to treatment [68].

Several limitations should be considered. We excluded visits that included diagnoses related to both opioid and stimulant use. Rates of drug-related ED visits and calendar time trends in rates could differ from our findings when including visits related to polysubstance use. The transition from *ICD-9-CM* to *ICD-10-CM *starting in 2015 potentially affected trends, as increasing rates of drug-related visits during this period could be attributed to this transition [69]. However, the continued rise across all drug-related ED visits provides some reassurance of validity. Use of diagnostic coding may not have accurately captured all-drug related ED visits, as it often relies on the judgment of the treating physician and the clinical presentation without confirmatory urine drug testing, which may introduce misclassification. We are also unable to rule out polysubstance use involvement even with coding for specific drug-related illnesses. Moreover, the data do not allow us to readily distinguish acute toxicity from withdrawal presentations (e.g., opioid-related visits treated with benzodiazepines and opioids are likely related to withdrawal). However, these preliminary data are needed to set the stage for longitudinal prospective studies to validate findings. Finally, we assume that the majority of psychostimulant-related visits were due to methamphetamine use, as psychostimulant-related *ICD* codes have high positive predictive values for methamphetamine use in acute care settings [2, 6, 7, 70]. However, we could not distinguish between visits related to methamphetamine versus other psychostimulants.

## Conclusions

Psychostimulant-related ED visits increased substantially from 2008 to 2018. Cocaine- and psychostimulant-related ED visits differ in presentation and management from opioid-related ED visits. They are less often identified as related to drug toxicity/withdrawal and require more interventions to address cardiopulmonary and psychiatric complications.

## Supplementary Information

**Additional file 1.**

## Data Availability

All data is made publicly available through the National Center of Health Statistics and can be accessed through their website at https://www.cdc.gov/nchs/ahcd/datasets_documentation_related.htm

## Abbreviations

ED: Emergency department
ICD: International classification of diseases
NCHS: National Center of Health Statistics
NHAMCS: National Hospital Ambulatory Medical Care Survey
RFV: Reason for visit
