# Supplementary material for: Emergency department visits and trends related to cocaine, psychostimulants, and opioids in the United States, 2008–2018
Source: BMC Emerg Med. 2022 Feb 4;22:19. doi: 10.1186/s12873-022-00573-0 (PMC8814795; doi:10.1186/s12873-022-00573-0)
Supplement: Supplementary file 1 — Additional file 1. [file 12873_2022_573_MOESM1_ESM.docx]

**Appendix 1. ICD-9-CM and ICD-10-CM diagnoses included for drug-related Emergency Department visits**

| **Substance** | **Variable** | **ICD9** | **ICD9 Description** | **ICD10** | **ICD10 Description** |
| --- | --- | --- | --- | --- | --- |
| Cocaine | coc | 304.20 | Cocaine dependence, unspecified | T40.5- | Poisoning by, adverse effect of and underdosing of cocaine |
|  |  | 304.21 | Cocaine dependence, continuous | F14- | Cocaine related disorders |
|  |  | 304.22 | Cocaine dependence, episodic | F14.1 | Cocaine abuse |
|  |  | 305.60 | Cocaine abuse, unspecified | F14.2 | Cocaine dependence |
|  |  | 305.61 | Cocaine abuse, continuous | F14.9 | Cocaine use, unspecified |
|  |  | 305.62 | Cocaine abuse, episodic |  |  |
|  |  | 970.81 | Poisoning by cocaine |  |  |
| Psychostimulants | stim | 304.40 | Amphetamine dependence, unspecified | F15- | Other stimulant related disorders |
|  |  | 304.41 | Amphetamine dependence, continuous | F15.1 | Other stimulant abuse |
|  |  | 304.42 | Amphetamine dependence, episodic | F15.2 | Other stimulant dependence |
|  |  | 305.70 | Amphetamine or related acting sympathomimetic abuse, unspecified | F15.9 | Other stimulant use, unspecified |
|  |  | 305.71 | Amphetamine or related acting sympathomimetic abuse, continuous | T43.6 | Poisoning by, adverse effect of and underdosing of unspecified psychostimulant |
|  |  | 305.72 | Amphetamine or related acting sympathomimetic abuse, episodic | T50.7 | Poisoning by analeptics and opioid receptor antagonists |
|  |  | 969.70 | Poisoning by psychostimulants NOS |  |  |
|  |  | 969.72 | Poisoning by amphetamine |  |  |
|  |  | 969.73 | Poisoning by methylphenidate |  |  |
|  |  | 969.79 | Poisoning by other psychostimulants |  |  |
|  |  | 970.0 | Poisoning by analeptics |  |  |
|  |  | 970.89 | Poisoning by other central nervous system stimulants |  |  |
|  |  | 970.9 | Poisoning by unspecified central nervous system stimulant |  |  |
|  |  | E854.2 | Accidental poisoning by psychostimulants |  |  |
|  |  | E854.3 | Accidental poisoning by central nervous system stimulants |  |  |
|  |  | E940.0 | Central nervous stimulants causing adverse effects in therapeutic use |  |  |
|  |  | E940.8 | Adverse effect of CNS stimulant, not elsewhere classified |  |  |
|  |  | E940.9 | Adverse effect of CNS stimulant, NOS |  |  |
| Opioids | opi | 292.0 | Drug withdrawal | F11- | Opioid-related disorders |
|  |  | 304.0 | Opioid type dependence | F11.1 | Opioid abuse |
|  |  | 304.00 | Opioid dependence, unspecified | F11.2 | Opioid dependence |
|  |  | 304.01 | Opioid dependence, continuous | F11.9 | Opioid use, unspecified |
|  |  | 304.02 | Opioid dependence, episodic | T40.0- | Poisoning by, adverse effect of, and underdosing of opium |
|  |  | 304.70 | Combinations of opioid type drug with any other drug dependence, unspecified | T40.1- | Poisoning by and adverse effect of heroin |
|  |  | 304.71 | Combinations of opioid type drug with any other drug dependence, continuous | T40.2- | Poisoning by, adverse effect of and underdosing of other opioids |
|  |  | 304.72 | Combinations of opioid type drug with any other drug dependence, episodic | T40.3- | Poisoning by, adverse effect of and underdosing of methadone |
|  |  | 305.50 | Opioid abuse, unspecified | T40.4- | Poisoning by, adverse effects of and underdosing of other synthetic narcotics |
|  |  | 305.51 | Opioid abuse, continuous | T40.6- | Poisoning by, adverse effect of and underdosing of other and unspecified narcotics |
|  |  | 305.52 | Opioid abuse, episodic | O99.31 | Alcohol use complicating pregnancy, childbirth, and the puerperium |
|  |  | 965.00 | Poisoning by opium (alkaloids), unspec |  |  |
|  |  | 965.01 | Poisoning by heroin |  |  |
|  |  | 965.02 | Poisoning by methadone |  |  |
|  |  | 965.09 | Poisoning by other opiates and related narcotics |  |  |
|  |  | E850.1 | Accidental poisoning by methadone |  |  |
|  |  | E850.2 | Accidental poisoning by other opiates and related narcotics |  |  |
|  |  | E935.1 | Methadone causing adverse effects in therapeutic use |  |  |
|  |  | E935.2 | Other opiates and related narcotics causing adverse effects in therapeutic use |  |  |
| Alcohol | alc | 291.0 | Alcohol withdrawal delirium | F10- | Alcohol related disorders |
|  |  | 291.1 | Alcohol-induced persisting amnestic disorder | F10.1 | Alcohol abuse |
|  |  | 291.2 | Alcohol-induced persisting dementia | F10.2 | Alcohol dependence |
|  |  | 291.3 | Alcohol-induced psychotic disorder with hallucinations | F10.9 | Alcohol use, unspecified |
|  |  | 291.4 | Idiosyncratic alcohol intoxication | T51.0 | Toxic effect of ethanol |
|  |  | 291.5 | Alcohol-induced psychotic disorder with delusions | T51.1 | Toxic effect of methanol |
|  |  | 291.81 | Alcohol withdrawal | T51.8 | Toxic effect of other alcohols |
|  |  | 291.82 | Alcohol induced sleep disorders | T51.9 | Toxic effect of unspecified alcohol |
|  |  | 292.89 | Other alcohol-induced mental disorders |  |  |
|  |  | 292.9 | Unspecified alcohol-induced mental disorders |  |  |
|  |  | 303.00 | Acute alcoholic intoxication in alcoholism, unspecified |  |  |
|  |  | 303.01 | Acute alcoholic intoxication in alcoholism, continuous |  |  |
|  |  | 303.02 | Acute alcoholic intoxication in alcoholism, episodic |  |  |
|  |  | 303.90 | Other and unspecified alcohol dependence, unspecified |  |  |
|  |  | 303.91 | Other and unspecified alcohol dependence, continuous |  |  |
|  |  | 303.92 | Other and unspecified alcohol dependence, episodic |  |  |
|  |  | 305.00 | Alcohol abuse, unspecified |  |  |
|  |  | 305.01 | Alcohol abuse, continuous |  |  |
|  |  | 305.02 | Alcohol abuse, episodic |  |  |
|  |  | 980.8 | Toxic effect of alcohol NEC |  |  |
|  |  | 980.9 | Toxic effect of alcohol NOS |  |  |
|  |  | E860.0 | Accidental poisoning by alcoholic beverages |  |  |
|  |  | E860.1 | Accidental poisoning by other and unspecified ethyl alcohol and its products |  |  |
|  |  | E860.9 | Accidental poisoning by unspecified alcohol |  |  |
| Cannabis | cann | 304.30 | Cannabis dependence, unspecified | F12- | Cannabis-related disorders |
|  |  | 304.31 | Cannabis dependence, continuous | F12.1 | Cannabis abuse |
|  |  | 304.32 | Cannabis dependence, episodic | F12.2 | Cannabis dependence |
|  |  | 305.20 | Cannabis abuse, unspecified | F12.9 | Cannabis use, unspecified |
|  |  | 305.21 | Cannabis abuse, continuous | T40.7- | Poisoning by, adverse effect of and underdosing of cannabis (derivatives) |
|  |  | 305.22 | Cannabis abuse, episodic |  |  |
| Hallucinogens | hallu | 304.50 | Hallucinogen dependence, unspec | F16- | Hallucinogen-related disorders |
|  |  | 304.51 | Hallucinogen dependence, continuous | F16.1 | Hallucinogen abuse |
|  |  | 304.52 | Hallucinogen dependence, episodic | F16.2 | Hallucinogen dependence |
|  |  | 305.30 | Hallucinogen abuse, unspecified | F16.9 | Hallucinogen use, unspecified |
|  |  | 305.31 | Hallucinogen abuse, continuous | T40.8- | Poisoning by and adverse effect of LSD |
|  |  | 305.32 | Hallucinogen abuse, episodic | T40.9- | Poisoning by, adverse effect of and underdosing of other and unspecified psychodysleptics (hallucinogens) |
|  |  | 969.6 | Poisoning by psychodysleptics (hallucinogens) |  |  |
|  |  | E854.1 | Accidental poisoning by psychodysleptics (hallucinogens) |  |  |
|  |  | E939.6 | Psychodysleptics (hallucinogens) causing adverse effects in therapeutic use |  |  |
| Sedative/  hypnotics | sed | 304.10 | Sedative, hypnotic or anxiolytic dependence, NOS | F13- | Sedative, hypnotic, or anxiolytic related disorders |
|  |  | 304.11 | Sedative, hypnotic or anxiolytic dependence, continuous | F13.1 | Sedative, hypnotic, or anxiolytic-related abuse |
|  |  | 304.12 | Sedative, hypnotic or anxiolytic dependence, episodic | F13.2 | Sedative, hypnotic, or anxiolytic-related dependence |
|  |  | 305.40 | Sedative, hypnotic or anxiolytic abuse, unspecified | F13.3 | Sedative, hypnotic, or anxiolytic-related use, unspecified |
|  |  | 305.41 | Sedative, hypnotic or anxiolytic abuse, continuous | T42.6X1 | Poisoning by other antiepileptic and sedative-hypnotic drugs, accidental (unintentional) |
|  |  | 305.42 | Sedative, hypnotic or anxiolytic abuse, episodic | T42.6X2 | Poisoning by other antiepileptic and sedative-hypnotic drugs, intentional self-harm |
|  |  | 967.6 | Poisoning by mixed sedatives, NEC | T42.6X3 | Poisoning by other antiepileptic and sedative-hypnotic drugs, assault |
|  |  | 967.8 | Poisoning by other sedatives and hypnotics | T42.6X4 | Poisoning by other antiepileptic and sedative-hypnotic drugs, undetermined |
|  |  | 967.9 | Poisoning by unspecified sedative or hypnotic | T42.6X5 | Adverse effect of other antiepileptic and sedative-hypnotic drugs |
|  |  | 969.4 | Poisoning by benzodiazepine-based tranquilizers | T42.6X2 | Underdosing of other antiepileptic and sedative-hypnotic drugs |
|  |  | E852.5 | Accidental poisoning by mixed sedatives, not elsewhere classified | T42.71X | Poisoning by, adverse effect of and underdosing of antiepileptic, sedative- hypnotic and antiparkinsonism drugs, accidental |
|  |  | E852.8 | Accidental poisoning by other specified sedatives, not elsewhere classified | T42.72X | Poisoning by, adverse effect of and underdosing of antiepileptic, sedative- hypnotic and antiparkinsonism drugs, intentional self-harm |
|  |  | E852.9 | Accidental poisoning by unspecified sedative or hypnotic | T42.73X | Poisoning by, adverse effect of and underdosing of antiepileptic, sedative- hypnotic and antiparkinsonism drugs, assault |
|  |  | E853.2 | Accidental poisoning by tranquilizers | T42.74X | Poisoning by, adverse effect of and underdosing of antiepileptic, sedative- hypnotic and antiparkinsonism drugs, undetermined |
|  |  | E939.4 | Benzodiazepine-based tranquilizers causing adverse effects in therapeutic use | T42.75X | Adverse effect of unspecified antiepileptic and sedative-hypnotic drugs |
|  |  | E980.2 | Poisoning by other sedatives and hypnotics, undetermined whether accidentally or purposely inflicted | T42.76X | Underdosing of unspecified antiepileptic and sedative-hypnotic drugs |
| Other or unspec drugs | othdrugs | 292.11 | Drug-induced psychotic disorder with delusions | F19- | Other psychoactive substance related disorders |
|  |  | 292.12 | Drug-induced psychotic disorder with hallucinations | F19.1 | Other psychoactive substance abuse |
|  |  | 292.2 | Pathological drug intoxication | F19.2 | Other psychoactive substance dependence |
|  |  | 292.81 | Drug-induced delirium | F19.9 | Other psychoactive substance use, unspecified |
|  |  | 292.82 | Drug-induced persisting dementia | T50.9- | Poisoning by, adverse effect of and underdosing of other and unspecified drugs, medicaments and biological substances |
|  |  | 292.83 | Drug-induced persisting amnestic disorder | O99.32 | Drug use complicating pregnancy, childbirth, and the puerperium |
|  |  | 292.84 | Drug-induced mood disorder |  |  |
|  |  | 292.85 | Drug-induced sleep disorders |  |  |
|  |  | 292.89 | Other specified drug-induced mental disorders |  |  |
|  |  | 292.9 | Unspecified drug-induced mental disorder |  |  |
|  |  | 304.60 | Other specified drug dependence, unspecified |  |  |
|  |  | 304.61 | Other specified drug dependence, continuous |  |  |
|  |  | 304.62 | Other specified drug dependence, episodic |  |  |
|  |  | 304.80 | Combinations of drug dependence excluding opioid type drug, unspecified |  |  |
|  |  | 304.81 | Combinations of drug dependence excluding opioid type drug, continuous |  |  |
|  |  | 304.82 | Combinations of drug dependence excluding opioid type drug, episodic |  |  |
|  |  | 304.90 | Unspecified drug dependence, unspecified |  |  |
|  |  | 304.91 | Unspecified drug dependence, continuous |  |  |
|  |  | 304.92 | Unspecified drug dependence, episodic |  |  |
|  |  | 305.90 | Other, mixed, or unspecified drug abuse, unspecified |  |  |
|  |  | 305.91 | Other, mixed, or unspecified drug abuse, continuous |  |  |
|  |  | 305.92 | Other, mixed, or unspecified drug abuse, episodic |  |  |
|  |  | 648.30 | Drug dependence of mother complicating pregnancy childbirth or puerperium, unspecified |  |  |
|  |  | 648.31 | Drug dependence of mother, delivered, with or without mention of antepartum condition |  |  |
|  |  | 648.32 | Drug dependence of mother, delivered, with mention of postpartum complication |  |  |
|  |  | 648.33 | Drug dependence of mother, antepartum condition or complication |  |  |
|  |  | 648.34 | Drug dependence of mother, postpartum condition or complication |  |  |
|  |  | E980.4 | Poisoning by other specified drugs and medicinal substances, undetermined whether accidentally or purposely inflicted |  |  |
|  |  | E980.5 | Poisoning by unspecified drug or medicinal substance, undetermined whether accidentally or purposely inflicted |  |  |

**Appendix 2. Reasons for visits codes for grouping of psychiatric, neurologic, cardiopulmonary, and drug toxicity/withdrawal chief presenting concerns**

| **RFV Group** | **Variable** | **RFV** | **RFV Code Title** |
| --- | --- | --- | --- |
| Cardiopulmonary | cardresp | 1030.0 | Fainting (syncope) |
|  |  | 1035.1 | Edema |
|  |  | 1050.0 | Chest pain and related symptoms |
|  |  | 1050.1 | Chest pain |
|  |  | 1050.2 | Chest discomfort pressure tightness |
|  |  | 1050.3 | Burning sensation in chest |
|  |  | 1260.0 | Abnormal pulsations and palpitations |
|  |  | 1260.1 | Increased heartbeat |
|  |  | 1260.2 | Decreased heartbeat |
|  |  | 1260.3 | Irregular heartbeat |
|  |  | 1263.0 | Heart pain |
|  |  | 1270.0 | Other symptoms of heart |
|  |  | 1280.0 | Other symptoms ref to the cardiovascular system |
|  |  | 1280.1 | Poor circulation |
|  |  | 1135.5 | Sleep apnea |
|  |  | 1415.0 | Shortness of breath |
|  |  | 1420.0 | Labored or difficult breathing (dyspnea) |
|  |  | 1425.0 | Wheezing |
|  |  | 1430.1 | Breathing problems NEC |
|  |  | 1430.2 | Disorders of respiratory sound NEC |
|  |  | 1440.0 | Rapid breathing (hyperventilation) |
|  |  | 1470.0 | Cough |
|  |  | 1485.0 | Other symptoms ref to respiratory system NEC |
|  |  | 2505.0 | Hypertension with involvement of targeted organs |
|  |  | 2510.0 | Hypertension |
|  |  | 2515.0 | Ischemic heart disease |
|  |  | 2520.0 | Other heart disease |
|  |  | 2350.0 | Atherosclerosis |
|  |  | 2550.0 | Other disease of circulatory system |
|  |  | 4401.0 | CPR |
|  |  | 5837.0 | Respiratory arrest |
|  |  | 5839.0 | Cardiopulmonary arrest |
|  |  | 5840.0 | Unconscious on arrival |
| Psychiatric | psych | 1100.0 | Anxiety and nervousness |
|  |  | 1105.0 | Fears and phobias |
|  |  | 1110.0 | Depression |
|  |  | 1115.0 | Anger |
|  |  | 1120.0 | Problems with identity and self-esteem |
|  |  | 1125.0 | Restlessness |
|  |  | 1130.0 | Behavioral disturbances |
|  |  | 1130.1 | Antisocial behavior |
|  |  | 1130.2 | Hostile behavior |
|  |  | 1130.3 | Hysterical behavior |
|  |  | 1130.4 | Temper problems |
|  |  | 1130.5 | Obsessions and compulsions |
|  |  | 1135.0 | Disturbances of sleep |
|  |  | 1135.1 | Insomnia |
|  |  | 1135.2 | Sleepiness (hypersomnia) |
|  |  | 1135.3 | Nightmares |
|  |  | 1135.4 | Sleepwalking |
|  |  | 1155.0 | Delusions or hallucinations |
|  |  | 1160.0 | Psychosocial disorders |
|  |  | 1165.0 | Other problems/symptoms related to psychological disorders |
|  |  | 2300.0 | Organic psychoses |
|  |  | 2310.0 | Neuroses |
|  |  | 2315.0 | Personality and character disorders |
|  |  | 2330.0 | Other and unspecified mental disorders |
|  |  | 2330.1 | ADD |
|  |  | 5818.0 | Intentional self-mutilation |
|  |  | 5820.0 | Suicide attempt |
| Neurologic | neuro | 1020.0 | General weakness |
|  |  | 1095.0 | Disorders of motor function |
|  |  | 1200.0 | Abnormal involuntary movements |
|  |  | 1205.0 | Convulsions |
|  |  | 1207.0 | Symptoms of head NEC |
|  |  | 1210.0 | Headache pain in head |
|  |  | 1215.0 | Memory disturbances of |
|  |  | 1220.0 | Disturbances of sensation |
|  |  | 1220.1 | Loss of feeling (anesthesia) |
|  |  | 1220.2 | Increased sensation (hyperesthesia) |
|  |  | 1220.3 | Abnormal sensation (paresthesia) |
|  |  | 1220.4 | Other disturbances of sense |
|  |  | 1225.0 | Vertigo – dizziness |
|  |  | 1230.0 | Weakness (neurologic) |
|  |  | 1235.0 | Disorders of speech, speech disturbance |
|  |  | 1235.1 | Stuttering stammering |
|  |  | 1235.2 | Slurring |
|  |  | 1240.0 | Other symptoms referable to the nervous system |
|  |  | 1305.0 | Vision dysfunctions |
|  |  | 1305.1 | Blindness and half vision |
|  |  | 1305.2 | Diminished vision |
|  |  | 1305.3 | Extraneous vision |
|  |  | 1305.4 | Double vision (diplopia) |
|  |  | 2360.0 | Epilepsy |
|  |  | 2365.0 | Migraine headache |
|  |  | 2370.0 | Other and unspecified disease of neurologic system |
|  |  | 2525.0 | Cerebrovascular disease |
|  |  | 5842.0 | Altered level of consciousness |
| Drug toxicity/withdrawal | drugs | 1140.0 | Smoking problems |
|  |  | 1145.0 | Alcohol-related problems |
|  |  | 1150.0 | Abnormal drug usage |
|  |  | 2320.0 | Alcoholism |
|  |  | 2321.0 | Drug dependence |
|  |  | 4518.0 | Detoxification |
|  |  | 4518.1 | Alcohol detoxification |
|  |  | 4518.2 | Drug detoxification |
|  |  | 5820.1 | Overdose, intentional |
|  |  | 5900.0 | Accidental poisoning |
|  |  | 5900.2 | Ingestion, inhalation, or exposure to potentially poisoning products |
|  |  | 5910.0 | Adverse effect of drug abuse |
|  |  | 5915.0 | Adverse effect of alcohol |
|  |  | 5916.0 | Alcohol poisoning |

**Appendix 3. Most common chief complaint among individuals presenting with opioid-, cocaine-, or psychostimulant-related ED visit, United States, 2008-2018**

| **Rank** | **Opioid-related visit** | **Freq %** | **Psychostimulant-related visit** | **Freq %** |
| --- | --- | --- | --- | --- |
| **1** | Adverse effect of drug abuse | 27.9 | Chest pain | 10.4 |
| **2** | Drug detoxification | 6.01 | Abnormal drug usage | 9.29 |
| **3** | Abnormal drug usage | 5.73 | Other symptoms related to psychoses | 8.66 |
| **4** | Organic psychoses | 5.61 | Anxiety and nervousness | 7.86 |
| **5** | Other symptoms related to psychoses | 5.32 | Shortness of breath | 7.59 |
| **6** | Detoxification | 5.01 | General psychiatric or psychological symptom | 7.35 |
| **7** | Cellulitis | 4.80 | Abdominal pain | 6.97 |
| **8** | Nausea | 4.05 | Adverse effect of drug abuse | 6.26 |
| **9** | Unconscious on arrival | 3.04 | Violence NOS | 4.88 |
| **10** | Overdose, intentional | 2.91 | Delusions or hallucinations | 4.80 |

| **Rank** | **Cocaine-related visit** | **Freq %** |
| --- | --- | --- |
| **1** | Chest pain | 27.2 |
| **2** | Other problems relating to psychoses | 7.88 |
| **3** | Abdominal pain | 5.73 |
| **4** | Adverse effect of drug abuse | 4.79 |
| **5** | Abnormal drug usage | 4.56 |
| **6** | Adverse effect of alcohol | 4.17 |
| **7** | Shortness of breath | 3.98 |
| **8** | Delusions or hallucinations | 3.97 |
| **9** | Overdose, intentional | 3.88 |
| **10** | General psychiatric or psychological symptoms | 3.45 |
